# Long Term Experience in Patients With JIA-Associated Uveitis in a Large Referral Center

**DOI:** 10.3389/fped.2021.682327

**Published:** 2021-06-28

**Authors:** Luca Marelli, Micol Romano, Irene Pontikaki, Maurizio Virgilio Gattinara, Paolo Nucci, Rolando Cimaz, Elisabetta Miserocchi

**Affiliations:** ^1^Eye Clinic San Giuseppe Hospital, Istituto di Ricovero e Cura a Carattere Scientifico (IRCCS) Multimedica, Milan, Italy; ^2^Pediatric Rheumatology Unit, Azienda Socio-Sanitaria Territoriale (ASST) G. Pini-Centro Traumatologico Ortopedico (CTO), Milan, Italy; ^3^Department of Clinical Sciences and Community Health, University of Milan, Milan, Italy; ^4^Department of Ophthalmology, San Raffaele Scientific Institute, University Vita-Salute, Milan, Italy

**Keywords:** JIA-associated uveitis, juvenile idiopathic arthritis, pediatric uveitis, safety, ocular complications

## Abstract

**Objectives:** To describe demographic, clinical and therapeutic findings of a large cohort of patients with JIA-associated uveitis in a nationwide referral pediatric rheumatology and uveitis center in Northern Italy.

**Methods:** Retrospective study of 125 patients with JIA-associated uveitis followed from 2009 to 2019. Demographic and rheumatologic features including JIA ILAR classification, age at onset, and laboratory data were recorded. Ocular findings collected were: anatomic location of uveitis, laterality, type, recurrence rate, visual acuity, ocular complications, and local therapy. Systemic therapy with conventional and biologic immunosuppressants, occurrence of adverse events, and duration of treatments were recorded.

**Results:** One hundred and twenty-five patients with JIA-associated uveitis were followed for a meantime of 9.2 (±1.7) years. Oligoarticular JIA was present in 92.8% of patients and anterior uveitis in 96%. The most common ocular complications recorded in our sample were posterior synechiae (37.6%), cataract (20.8%), band keratopathy (19.2%), glaucoma (7.2%), and macular edema (5.6%). Conventional immunosuppressants were used in 75.2% of patients with a mean duration of 9.1 years (±5.4), while biologics were administered in 47.2% of them for a period of 5.4 years. Adverse events (AE) were seen in 23% of patients being treated with Methotrexate, in 10.4% of patients treated with Adalimumab, in 38.5% of patients in therapy with Infliximab, and in 14.3% of patients being treated with Tocilizumab. No AE were reported in patients treated with Golimumab, Certolizumab, Abatacept and Rituximab.

**Conclusions:** An aggressive treatment approach for patients with JIA-associated uveitis ensured a low number of ocular complications with a good safety profile.

## Introduction

Juvenile idiopathic arthritis (JIA) accounts for ~75% of all pediatric anterior uveitis cases. JIA is the most common chronic pediatric rheumatic disease with an annual incidence of 2–20 per 100,000 children, and a prevalence of 16–150 per 100,000 (1, 2).

Children with JIA associated uveitis usually present with an insidious and asymptomatic chronic anterior uveitis that in ~25–50% of cases may lead to severe vision-threatening complications such as synechiae, cataracts, and glaucoma when not successfully controlled by therapy (3).

Vision-related quality of life of patients with JIA-associated uveitis has been described as poor, because of high incidence of visual impairment, which impacts on a patient's capability to perform visual tasks and daily activities (4). Moreover, due to the chronic course of this disease, children undergo periodic ophthalmologic and rheumatologic evaluations and need long-term immunomodulatory treatments (5).

Despite diagnostic and therapeutic advances in the past decades, JIA-associated uveitis still represents a critical health issue because of the lifelong duration of the disease and the high risk of ocular morbidity. Given the severity of the ocular and rheumatologic condition children with JIA should be followed in tertiary care centers where there is a multidisciplinary team of pediatric rheumatology specialists and a trained uveitis expert that cooperate to improve diagnostic and therapeutic issues of these patients. Adherence to regular ophthalmologic screening programs and interdisciplinary management appear to be the best way to prevent complications and reduce the risk of visual impairment.

The purpose of the present study is to provide demographic, clinical and therapeutic findings of a large cohort of patients with JIA-associated uveitis followed in a nationwide referral pediatric rheumatology and uveitis center in Northern Italy.

## Materials and Methods

### Patients

This is a retrospective analysis including 125 consecutive patients affected by JIA-associated uveitis seen at the pediatric rheumatology and uveitis unit, at the ASST G. Pini in Milano, a tertiary referral nationwide center in Northern Italy. In this hospital children and adolescents affected by JIA are regularly seen and referred from all other Italian regions.

**Figure 1:**
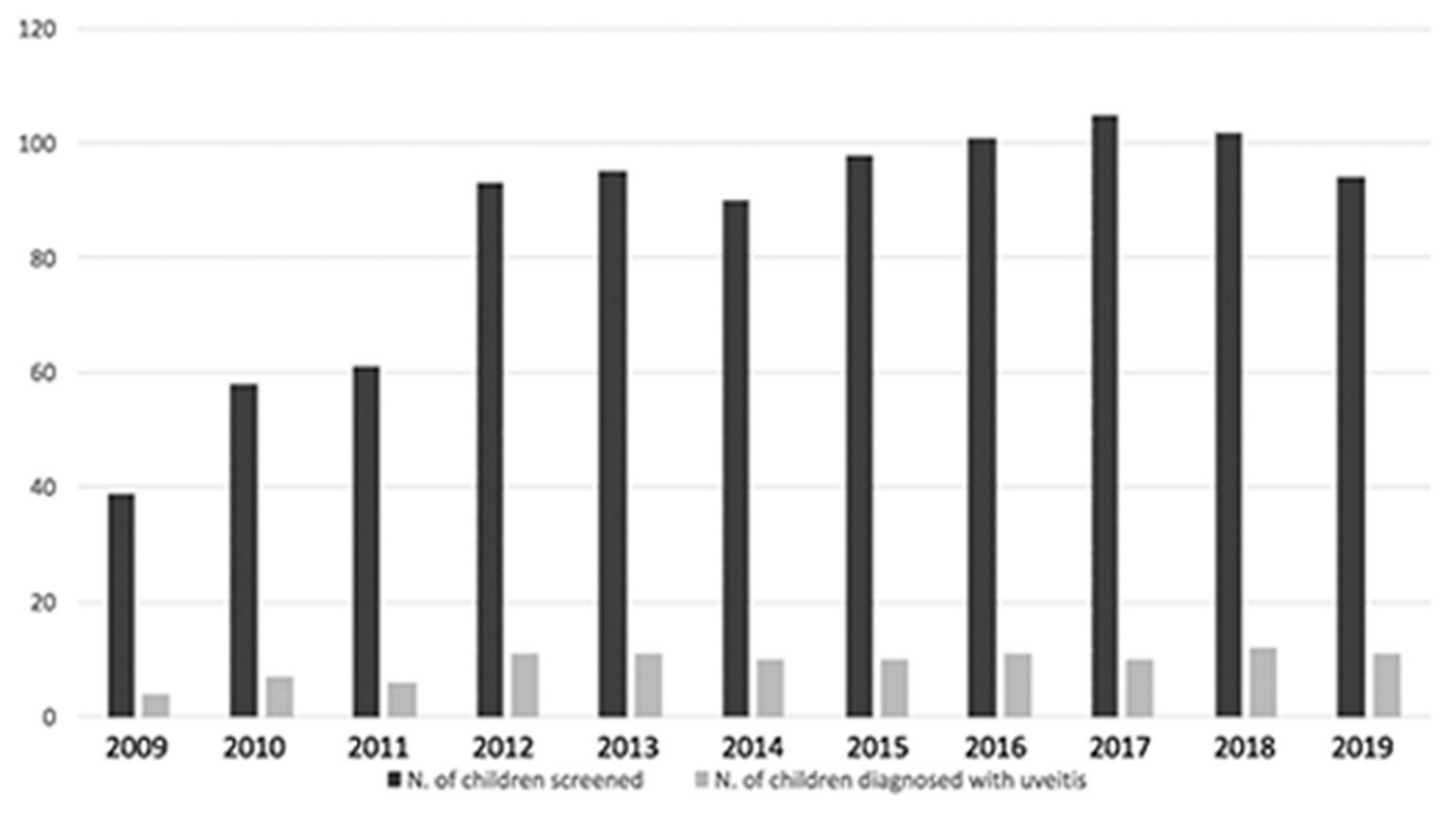
Bar graph showing distribution of patients with JIA screened and diagnosed with uveitis during eleven-year screening period.

From January 2009 to December 2019 6,802 screening visits of 936 children affected by JIA were performed and during this eleven-year screening period 103 children with JIA were diagnosed with uveitis and then followed-up in the uveitis service (Figure 1). Twenty-two additional patients were diagnosed in other centers and then referred to us. A total of 125 patients was recruited.

Informed consent was obtained from all parents and from patients older than 18 years of age, as required by the Italian bioethical legislation, in agreement with the Declaration of Helsinki for research involving human subjects.

### Methods

The diagnosis of JIA was made by a pediatric rheumatologist based on the ILAR classification criteria (6). Patients included in this study were required to have disease onset prior to 16 years, associated with unilateral or bilateral uveitis.

The following parameters were evaluated: gender, ethnicity, age at onset of arthritis, age at onset of uveitis, type of JIA classified by ILAR criteria, laboratory data including anti-nuclear antibody (ANA), rheumatoid factor (RF), anti-cyclic citrullinated peptide (CCP), activity of arthritis and systemic therapy.

All patients with a diagnosis of JIA-associated uveitis were regularly evaluated by the same trained uveitis specialist (EM). At each visit the ophthalmological evaluation included: best-corrected Snellen visual acuity, intraocular pressure measurement with Goldmann tonometry or Icare measurement, slit-lamp evaluation of anterior segment, dilated fundus examination and laser flare meter assessment. Fluorescein angiography, indocyanine green angiography and optical coherence tomography (OCT) were carried out when indicated in case of posterior pole involvement.

Clinical characteristics evaluated were: anatomic classification of uveitis, laterality, type of uveitis (granulomatous or non-granulomatous), activity of uveitis, rate of uveitis recurrences, presence of ocular complications, local and systemic treatment. Granulomatous uveitis was defined by the presence of at least one of the following: Busacca nodules, anterior chamber angle nodules, mutton-fat keratic precipitate (KP), or ghost KP at any point in the course of the disease, as reported in the literature (7).

Anatomic classification of uveitis (anterior, intermediate, posterior or panuveitis) was performed according to the Standardization of Uveitis Nomenclature Workshop (SUN) criteria (8). Anterior uveitis produces inflammatory signs localized primarily to the anterior segment of the eye, as a result of inflammation of the iris and pars plicata. In intermediate uveitis, inflammation occurring in the ciliary body, pars plana and/or peripheral retina is most prominent in the vitreous cavity. In posterior uveitis intraocular inflammation primarily involves the retina and/or choroid and fundus oculi exam reveals focal, multifocal, or diffuse areas of retinitis and/or choroiditis, often with retinal vasculitis. In panuveitis, inflammation involves anterior, intermediate and posterior uveal structures without a predominantly affected site. Inflammatory activity was graded according to the SUN criteria for the anterior (cells and flare) and posterior (vitreous cells and haze) chambers, from grade 0 to 4. Semi-automated flare measurement with the Kowa FC500 Laser Flare Meter (LFM) was employed for flare quantification. Improvement was defined as a reduction of anterior chamber cells (two-step decrease) in the level of inflammation or a reduction of flare by means of LFM (under 50 ph/ms). In addition, a macular scan by means of optical coherence tomography (OCT; Spectralis, Spectral domain-OCT; Heildelberg Engineering, Germany) was performed in patients with posterior uveitis and macular edema. Improvement was defined as a resolution of macular edema on OCT (under 300 μm).

Ocular complications including the presence of band keratopathy, posterior synechiae, cataract, glaucoma, macular edema and other posterior pole abnormalities were recorded. Surgical interventions such as cataract extraction, glaucoma filtering surgery and other type of surgeries during follow-up were analyzed.

The frequency of ophthalmological evaluations performed by our trained uveitis specialist (EM) ranged from once a week to every 2–3 months according to the severity and course of the uveitis.

### Treatment Protocol

Acute or new onset uveitis was treated with mydriatics and topical corticosteroids according to the degree of intraocular inflammation and then tapered during a course of 3 months.

Oral systemic corticosteroids were added in case of posterior pole involvement and in the presence of macular edema.

In case of persistent or recurrent activity of anterior chamber inflammation or in case of steroid-dependency, patients were started with systemic disease modifying anti-rheumatic drugs (DMARDs). Our first line immunosuppressive treatment was Methotrexate (MTX) at 10–15 mg/m^2^/week given orally or subcutaneously in case of gastrointestinal intolerance.

Systemic cyclosporine (CSA) at 3–5 mg/Kg/day was prescribed as a second line conventional DMARDs in case of intolerance to methotrexate.

Biologic DMARDs (b-DMARDs) were prescribed in case of persistence of intraocular inflammation despite systemic MTX or CSA. All patients underwent a protein-purified derivative (PPD) skin test and chest radiograph before initiation of biologic therapy. Tumor Necrosis Factor-α (TNF-α) blockers were the first line biologics used in JIA-associated uveitis.

Adalimumab was given subcutaneously every 2 weeks at a dosage of 24 mg/m^2^ (maximum dosage 40 mg), Infliximab was administered intravenously at a dosage of 6 mg/Kg (scheduled at 0, 2 and monthly thereafter). Other anti-TNF-α agents such as Golimumab (40 mg every 4 weeks) and Certolizumab Pegol (150 mg) were prescribed in case of failure or intolerance.

In cases of persistently active uveitis and/or arthritis patients were swapped to other classes of b-DMARDs: Rituximab (375 mg/m^2^), Tocilizumab (8 mg/kg), Abatacept (10 mg/kg). JAK-inhibitors were used in 2 patients (9).

Treatment-related adverse events were recorded for each patient during follow-up.

All data were collected in a protected and anonymized database and processed using commercial software (SPSS for Windows version 20; SPSS Sciences, Chicago, IL). Ethics committee was not deemed necessary since according to local regulation anonymized data were retrieved.

## Results

**Figure 2:**
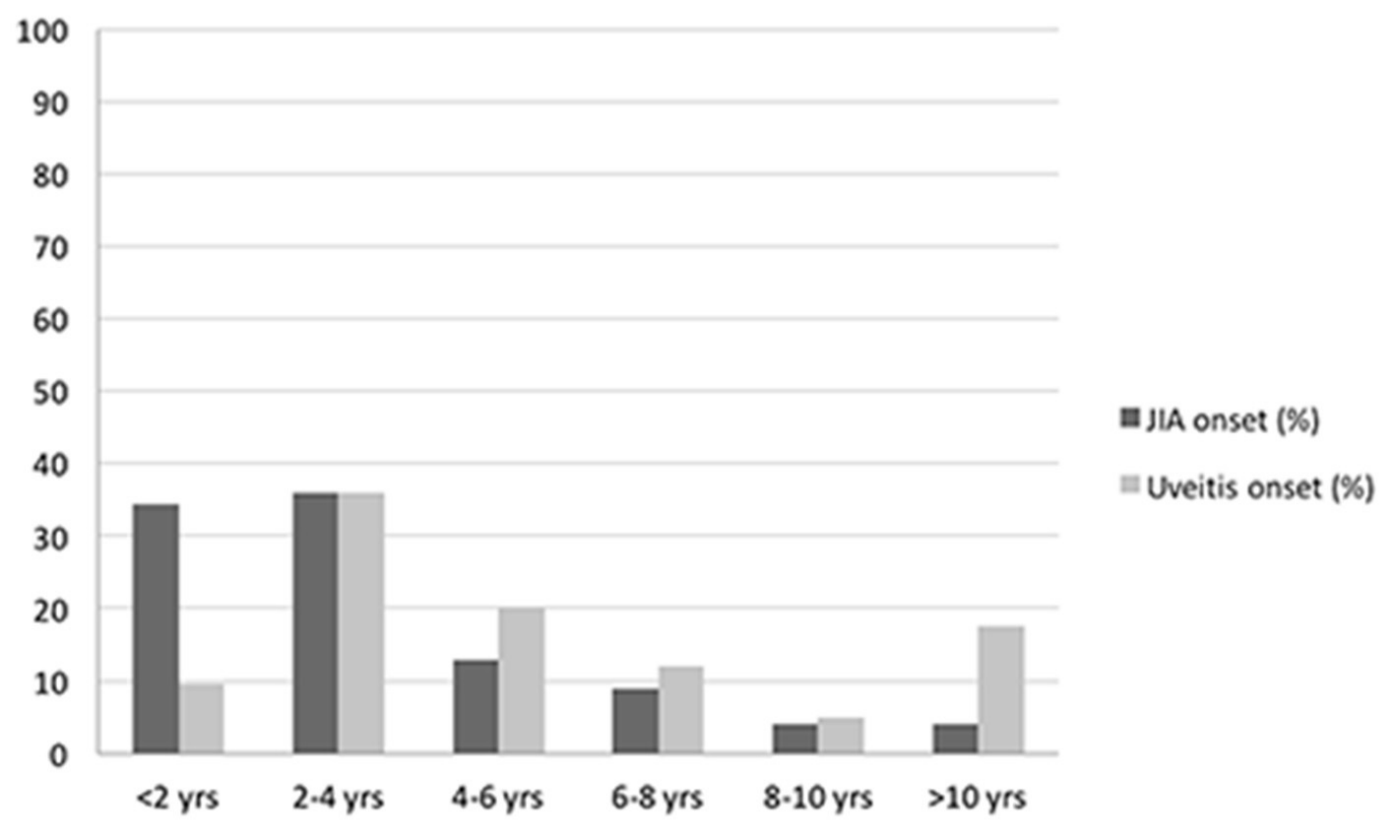
Bar graph showing age distributions of patients at diagnosis of JIA and uveitis.

A total of 125 patients diagnosed with JIA-associated uveitis was included into the study group, with a mean (±SD) follow-up of 9.2 years (±1.7). Among these patients, 100 were female (80.0%) and 25 were male (20.0%) with a female-to-male ratio of 4:1. Mean (±SD) age at diagnosis of JIA was 3.5 years (±3.1). Mean (±SD) duration of arthritis was 10.4 years (±5.1). Oligoarticular JIA was the most common subtype (92.8%), followed by polyarthritis (5.6%) and enthesitis related arthritis (1.6%). Positive ANA (titer of 1:160 or above) was present in 112 (89.6%) patients, while RF was negative in 100% of the polyarticular JIA subjects. Age distributions of patients at diagnosis of JIA and uveitis are represented in Figure 2. Mean (±SD) age at the time of uveitis diagnosis was 6.1 years (±5.4). Mean (±SD) intervel between JIA onset and uveitis diagnosis was 2.6 years (±4.7).

Among patients, 91 developed a non-granulomatous uveitis (72.8%), 34 a granulomatous type.

Most of subjects had anterior uveitis (120, 96.0%). Two patients had posterior uveitis (1.6%), and three patients had panuveitis (2.4%). Bilateral involvement was noticed in 84 subjects (67.2%).

Overall, 67 patients (53.6%) developed at least one ocular complication. Posterior synechiae were the most common, occurring in 37.6% of cases; cataracts, band keratopathy, glaucoma and macular edema were observed in 20.8, 19.2, 7.2, and 5.6% respectively, as shown in Table 1.

**Table 1:** Demographic and clinical features of our sample of patients.

**Mean age at the time of uveitis diagnosis** years (±SD)	6.1 (±5.4)
**Mean duration of uveitis** years (±SD)	8.9 (±2.0)
**Mean interval JIA-uveitis diagnosis** years (±SD)	2.6 (±4.7)
**Anatomic classification of uveitis**	
Anterior uveitis *N*. (%)	120 (96%)
Posterior uveitis *N*. (%)	2 (1.6%)
Panuveitis *N*. (%)	3 (2.4%)
**Laterality of uveitis**	
Unilateral *N*. (%)	41 (32.8%)
Bilateral *N*. (%)	84 (67.2%)
**Type of uveitis**	
Granulomatous *N*. (%)	34 (27.2%)
Non-granulomatous *N*. (%)	91 (72.8%)
**Complications** *N*. (%)	67 (53.6%)
Synechiae *N*. (%)	47 (37.6%)
Glaucoma *N*. (%)	9 (7.2%)
Cataract *N*. (%)	26 (20.8%)
Band keratopathy *N*. (%)	24 (19.2%)
Cystoid macular edema *N*. (%)	7 (5.6%)
**Glaucoma medical therapy** *N*. (%)	9 (7.2%)
**Surgery**	
Surgical management of band keratopathy *N*. (%)	1 (0.8%)
Synechiotomy *N*. (%)	1 (0.8%)
Cataract surgery *N*. (%)	23 (18.4%)
Glaucoma surgery *N*. (%)	1 (0.8%)
**Rate of recurrence** *N*. episodes per year (mean ± SD)	1.2 (±1.0)

Mean duration of uveitis was 8.9 years (±2.0) and rate of recurrence was 1.2 (±1.0) episodes per year.

Table 1 depicts the number and types of surgeries performed by patients, with 23 of them having undergone cataract surgery, one surgical removal of band keratopathy, synechiotomy and glaucoma filtering surgery.

Our study also provides an overview of the visual impairment showing the monocular best-corrected visual acuity (BCVA) as measured in decimal units at the end of the monitoring period. A BCVA ≤0.4 was observed in 16.0% (20/125) of patients (worst eye open only), while 16 out of 125 patients (12.8% of total sample) experienced a monocular BCVA ≤0.1 (worst eye open only). Among these patients with visual impairment, 5 (4%) had a monocular BCVA ≤0.4 in both eyes, while 3 (2.4%) of them experienced a monocular BCVA ≤0.1 in the two eyes.

With regard to treatment, 96 were on DMARDs (76.8%), and 59 on biologic therapy (47.2%) at final follow-up visit, and the mean treatment duration was 9.1 (±5.9), and 5.3 (±3.2) years, respectively. Out of 125 patients, only 6 were receiving systemic corticosteroids. Specifically, ongoing treatment at the end of monitoring period is summarized in Table 2.

**Table 2:** Systemic treatment at initial evaluation/during follow-up and at last evaluation.

**Treatment**	**Initial/during follow-up *N*. (%)**	**Drug discontinuation *N*. (%)**	**Final evaluation *N*. (%)**
**Systemic corticosteroids** (%)	57 (45.6%)	51 (40.8%)	6 (4.8%)
**DMARDs**			
Methotrexate (%)	100 (80.0%)	26 (20.8%)	74 (59.2%)
Cyclosporine (%)	24 (19.2%)	12 (9.6%)	12 (9.6%)
**Biological treatment** (%)			
Adalimumab (%)	48 (38.5%)	15 (12%)	33 (26.4%)
Infliximab (%)	26 (20.8%)	18 (14.4%)	8 (6.4%)
Certolizumab (%)	2 (1.6%)	1 (0.8%)	1 (0.8%)
Golimumab (%)	7 (5.6%)	2 (1.6%)	5 (4%)
Tocilizumab (%)	7 (5.6%)	4 (3.2%)	3 (2.4%)
Abatacept (%)	4 (3.2%)	2 (1.6%)	2 (1.6%)
Rituximab (%)	5 (4.0%)	2 (1.6%)	3 (2.4%)
**JAK-inhibitors** (%)			
Tofacitinib (%)	1 (0.8%)	-	1 (0.8%)
Baricitinib (%)	1 (0.8%)	-	1 (0.8%)

Among the 57 patients being treated with systemic steroids at baseline because of arthropathy (*n* = 55) or due to panuveitis at onset (*n* = 2), 51 (89.5%) were able to discontinue treatment, achieving corticosteroid-sparing control and reducing the risk for side effects.

Methotrexate was given to 100 patients (80%) during follow-up; of these, 26 subjects (26%) discontinued therapy due to adverse effects (23%), pregnancy wish (2%) and loss of efficacy (1%). Out of 48 patients being treated with Adalimumab, 15 (31.3%) discontinued it due to loss of efficacy over time (9/48, 18.8%), pregnancy wish (1/48, 2%) and adverse events (5/48, 10.4%).

Twenty-six patients (20.8%) were treated with Infliximab. Therapy was stopped in 18 of them (18/26, 69.2%). Reasons for discontinuation of Infliximab were loss of efficacy over time (7/26, 26.9%), pregnancy wish (1/26, 3.8%), and adverse events (10/26, 38.5%). No adverse events were reported in patients treated with other biologics including Golimumab, Certolizumab, Abatacept and Rituximab. The mean treatment time (±SD) with these drugs was 47.7 months (±38.7), 18 months (±1.4), 24.8 months (±19.9), 33 months (±23), respectively. Out of 7 patients being treated with Tocilizumab, 4 of them (57.2%) discontinued it due to loss of efficacy (3/7, 42.9%) and adverse reactions (1/7, 14.3%) (Table 3).

**Table 3:** Drug-related reasons for drug discontinuation.

**Reasons for drug discontinuation**	**MTX *N*. pts (%)**	**CSA*N*. pts (%)**	**ADA *N*. pts (%)**	**INF*N*. pts (%)**	**GOL *N*. pts (%)**	**CER*N*. pts (%)**	**TCZ *N*. pts (%)**	**ABA*N*. pts (%)**	**RTX *N*. pts (%)**
Loss of efficacy	1/100 (1%)	9/24 (37.5%)	9/48 (18.8%)	7/26 (26.9%)	2/7 (28.6%)	1/2 (50%)	3/7 (42.9%)	2/4 (50%)	2/5 (40%)
Pregnancy wish	2/100 (2%)	-	1/48 (2%)	1/26 (3.8%)	-	-	-	-	-
Liver toxicity	6/100 (6%)	-	-		-	-	-	-	-
Gastro-intestinal upset	15/100 (15%)	1/24 (4.2%)	-	3/26 (11.5%)	-	-	1/7 (14.3%)	-	-
Infections	1/100 (1%)	-	-		-	-	-	-	-
Allergic reaction	1/100 (1%)	-	-	1/26 (3.8%)	-	-	-	-	-
Dermatological	-	-			-	-	-	-	-
- Psoriasis			-	1/26 (3.8%)					
- Hydrosadenitis			1/48 (2%)	-					
- Lichen planus			-	1/26 (3.8%)					
- Alopecia			-	1/26 (3.8%)					
Injection site reaction	-	-	2/48 (4.2%)	3/26 (11.5%)	-	-	-	-	-
Psychological disturbances	-	-	2/48 (4.2%)	-	-	-	-	-	-
Hypertension	-	2/24 (8.3%)	-	-	-	-	-	-	-
Total	26/100	12/24	15/48	18/26	2/7	1/2	4/7	2/4	2/5

*MTX, Methotrexate; CSA, Cyclosporin A; ADA, Adalimumab; INF, Infliximab; GOL, Golimumab; CER, Certolizumab; TCZ, Tocilizumab; ABA, Abatacept; RTX, Rituximab*.

Moreover, 12 patients were on Etanercept treatment before the onset of uveitis. All of them (12/12, 100%) discontinued it because of loss of efficacy (6/12, 50.0%) onset of uveitis (4/12, 33.3%), diagnosis of Crohn's disease (1/12, 8.3%) and mood disorders (1/12, 8.3%).

Among 101 patients with available data, in 67.3% of cases uveitis started before immunosuppression, and in 32.7% of cases uveitis started during immunosuppression.

Out of 20 patients with visual impairment, 2 of them were treated with systemic steroids, 3 of them with DMARDs, 4 of them with 1 bDMARD, while 11 patients required more than 1 bDMARDs during follow-up period.

## Discussion

Among extra-articular complications of JIA, uveitis is the most frequent and severe (10), importantly affecting children's quality of life. In our cohort, the mean age at the time of uveitis diagnosis was 6.1 years, but most of the uveitis cases were identified between the age of 2 and 4 years old, with a mean interval of 2.6 years after the onset of arthritis.

These data are consistent with most of the literature reports, in which uveitis is diagnosed after the onset of arthritis (11), although in rare cases (3–7%) the uveitis has been reported to preceed the arthritis in variable range of years (12–14).

In our sample, the majority of children (88%) presented with arthritis before the uveitis onset, while the uveitis was diagnosed concurrently with the onset of arthritis in the remaining 12% of patients.

Anterior uveitis was present in 96% of our cohort, was bilateral in the majority of cases (67%) and non-granulomatous type of uveitis was the most frequent finding similarly to other literature reports (7). JIA-associated uveitis has typically been described as non-granulomatous (14, 15). However, after excluding other forms of granulomatous uveitis, such as sarcoidosis, JIA-associated uveitis should always be considered in the differential diagnosis of granulomatous uveitis, even in the absence of articular manifestations.

Ocular complications, possibly resulting from both the disease itself and its therapies, are frequently observed in JIA-associated uveitis. In the pediatric population, at risk for amblyopia, they could be particularly damaging. In our 9.2-year mean follow-up study involving 125 patients, the overall rate of ocular complications was 53.6% at last follow-up visit. Posterior synechia was the most frequently seen, occurring in 37.6% of patients, followed by cataract in 20.8%, band keratopathy in 19.2%, glaucoma 7.2%, and cystoid macular edema in 5.6%. Additionally, 20.8% of them required surgery (18.4% cataract surgery, 0.8% surgical removal of band keratopathy, 0.8% synechiotomy and 0.8% glaucoma surgery).

In a recent retrospective analysis including 108 patients with JIA-associated uveitis, the authors described the incidence of ocular hypertension and secondary glaucoma in 40% of eyes at presentation and a three-fold higher incidence of legal blindness during a 5-year median follow-up (16). Moreover, the authors found a correlation between the use of systemic corticosteroids at presentation and incidence of hypertension/secondary glaucoma, concluding that an early and aggressive immunosuppressive therapy may reduce the risk of developing such complications.

Macular edema was a rare complication in our series, occurring only in 5.6% of patients, a lower incidence compared to other studies (17) in which this posterior pole complication has been reported as 26.5% over a follow up of 11.6 years. A correlation between duration of uveitis and development of this sight threatening complication has been reported in this clinical study (17). Given the comparable follow-up between the two groups, these results could be partially explained by the different use of OCT imaging. In our cohort OCT scan was not performed in all children at every visit, but only in selected situations, therefore we may have missed cases of macular edema. This could be considered a limitation of our study.

Paroli et al. (18), in their retrospective cohort study set in an Italian tertiary referral center, described the frequencies of ocular complication in 69 consecutive children (116 eyes) affected by JIA-U. At baseline, 52% of patients presented with posterior synechiae, 38% with band keratopathy, and 12% with cataract. After a median follow-up of 38 months, 49 additional eyes (48%) developed cataract, 22 (19%) had elevated IOP (intraocular pressure), and 16 (14%) were diagnosed with macular edema. Additionally, 21 eyes required cataract surgery and 1 underwent glaucoma surgery in order to control the IOP.

Thorne et al. (19) estimated the incidences of ocular complications after collecting clinical data of 75 patients with JIA-associated uveitis. Of these, 43% received oral corticosteroid therapy, and in 60% of patients immunosuppressive drug therapy was administered at some point during the disease course, including methotrexate, cyclosporine, mycophenolate mofetil, tacrolimus, and chlorambucil. Since their analysis dates back to the year 2007, only three patients were treated with a biologic DMARD (infliximab and etanercept). Ocular hypertension was reported to be the most frequent, affecting 46.3% of the cohort, followed by band keratopathy (28.0%) and cataract (20.0%). Interestingly, despite that follow-up (median duration = 3 years) was shorter than ours, more children required surgery. In particular, 21.9% underwent glaucoma surgery, while cataract surgery was performed in 19.5% of the children.

Administration of corticosteroids for several months is known to be associated with an increased risk of cataract and glaucoma (20). The incidence of cataract has been evaluated in a series of 75 children with JIA-associated uveitis treated with different daily doses of topical corticosteroids; for eyes being administered steroids 3 times daily, the rate of cataract development was 0.01/EY (eye-year), while this rate for eyes treated topically 4 times daily it was 0.07/EY (21).

With the therapeutic approach previously described, we managed to discontinue systemic steroids in 89.5% of our patients, ensuring them corticosteroid-sparing control and reducing the risk for related complications.

Biologic DMARDs have largely demonstrated their efficacy in treatment of JIA-associated uveitis, potentially improving the further outcome of uveitis and preventing relapses (22–26). Given the long term follow-up, our study provides interesting data concerning safety and tolerability of these drugs. Out of 59 patients being treated with biologic DMARDs, only 16 of them discontinued due to adverse events. In a recent multicenter study conducted in Italy and including 154 patients treated with Infliximab or Adalimumab, Cecchin et al. reported no major adverse events during a 2-year follow-up, while 34 minor events, including infections, infusion reactions and GI symptoms were described (27).

Therefore, considering early onset, high incidence of sight-threatening complications and high risk for amblyopia of this population, a prompt care of these patients is required.

In our experience, the aggressive treatment approach with the “zero tolerance” philosophy for total eradication of intraocular inflammation has led to a low number of ocular complications during the long-term follow-up.

Children with JIA-associated uveitis seen in our tertiary referral center are treated with a long-term program of systemic immunosuppressive therapies, starting from conventional DMARDs and rapidly moving to different biologic DMARDs in case of uncontrolled uveitis, avoiding chronic corticosteroid usage. The long-term duration of treatment of our cohort ensured a good control of intraocular inflammation over time with reduced number of cataract and glaucoma and also a very low need for surgical intervention such as cataract (18.4%) and filtering surgery (0.8%).

Close collaboration and team-approach with pediatric rheumatologist is another important aspect in the care of our JIA uveitis patients. Treatment decisions, aggressiveness and close monitoring of systemic therapies by pediatric rheumatology unit has led to a limited number of overall treatment related side effects.

In conclusion, despite the retrospective and monocentric type of analysis, our study showed that JIA-associated uveitis patients followed in multidisciplinary center may benefit from long-term aggressive immunosuppressive therapy with negligible secondary side-effects.

## Data Availability Statement

The raw data supporting the conclusions of this article will be made available by the authors, without undue reservation.

## Ethics Statement

Ethical review and approval was not required for the study on human participants in accordance with the local legislation and institutional requirements. Written informed consent to participate in this study was provided by the participants' legal guardian/next of kin.

## Author Contributions

All authors gave substantial contributions to study conception and design, contributions to analysis, and interpretation of data.

## Conflict of Interest

The authors declare that the research was conducted in the absence of any commercial or financial relationships that could be construed as a potential conflict of interest.
